# Effects of Chokeberry Juice Added to a Mediterranean-Style Diet on Glycemic Control and Redox Homeostasis in Women with Prediabetes: A Randomized Controlled Trial

**DOI:** 10.3390/nu18182948

**Published:** 2026-09-09

**Authors:** Małgorzata Elżbieta Zujko, Marta Rożniata, Marta Żebrowska-Gamdzyk

**Affiliations:** 1 Department of Food Biotechnology, Faculty of Health Sciences, Medical University of Białystok, Szpitalna 37, 15-295 Białystok, Poland; 2 Department of Dietetics, Faculty of Health Sciences, University of Łomża, Akademicka 14, 18-400 Łomża, Poland; mrozniata@al.edu.pl (M.R.); mzebrowska@al.edu.pl (M.Ż.-G.)

**Keywords:** Mediterranean diet, polyphenols, antioxidants, prediabetes, prevention

## Abstract

**Background**: Prediabetes is characterized by impaired glucose regulation and altered redox homeostasis, which may contribute to progression to type 2 diabetes. Polyphenol-rich foods may improve metabolic and redox balance. **Methods**: Fifty-four women aged 30–60 years with prediabetes were randomly assigned to an intervention group (IG) or control group (CG); 50 completed the 12-week study (25 per group). Both groups received Mediterranean diet recommendations. The IG additionally consumed 100 mL/day of chokeberry juice, providing 457 mg GAE of total polyphenols and 9.7 mmol antioxidant capacity (FRAP), whereas the CG received no study product. Fasting plasma glucose (FG), HbA1c, 2 h plasma glucose (2h-PG), total oxidant status (TOS), total antioxidant status (TAS), and oxidative stress index (OSI) were assessed at baseline and 12 weeks. Between-group differences in individual changes were analyzed using the Mann–Whitney U test with Benjamini–Hochberg false discovery rate correction. The randomized substudy reported here was conducted within the broader single-group parent study registered at ClinicalTrials.gov (NCT06435130), which enrolled a larger mixed-sex cohort; the present analysis concerns only the nested 12-week randomized comparison in women with prediabetes. **Results**: Compared with the CG, the IG showed greater reductions in FG, HbA1c, 2h-PG, TOS, and OSI and a greater increase in TAS. All between-group differences remained significant after FDR correction (adjusted *p* < 0.001), with large Cliff’s delta values. **Conclusions**: Adding 100 mL/day of chokeberry juice to Mediterranean diet recommendations for 12 weeks was associated with modest but statistically significant improvements in glycemic and redox-related outcomes in women with prediabetes. The small sample, short follow-up, and lack of placebo control limit generalizability; larger, longer placebo-controlled trials are warranted.

## 1. Introduction

Prediabetes is an intermediate metabolic state between normoglycemia and type 2 diabetes mellitus (T2DM), characterized by impaired fasting glucose, impaired glucose tolerance, and/or elevated glycated hemoglobin (HbA1c) levels that do not meet the diagnostic criteria for diabetes. Its prevalence has been increasing worldwide and currently affects hundreds of millions of adults. Individuals with prediabetes are at increased risk of developing T2DM, cardiovascular disease, and other chronic conditions [1].

Oxidative stress plays an important role in the pathogenesis of T2DM. Contemporary redox biology recognizes that reactive oxygen species (ROS) are not merely harmful by-products of aerobic metabolism but also important signaling molecules involved in the regulation of cellular metabolism and maintenance of homeostasis [2]. Under physiological conditions, ROS-mediated signaling contributes to the regulation of glucose uptake, mitochondrial function, endogenous antioxidant defenses, and inflammatory responses. Chronic metabolic disturbances, however, may disrupt this tightly regulated redox balance, resulting in a pro-oxidant environment and impaired redox homeostasis. Pancreatic β-cells are particularly vulnerable to oxidative stress because of their relatively limited antioxidant defense capacity. Persistent oxidative stress may impair insulin secretion, reduce β-cell viability, and thereby contribute to the progression from prediabetes to overt T2DM [3,4].

Lifestyle modification remains the cornerstone of prediabetes management. Large intervention studies and current clinical recommendations indicate that improvements in diet quality, increased physical activity, and weight reduction can substantially reduce the risk of progression to T2DM [5,6]. Nevertheless, there is a need to identify specific nutritional strategies that may simultaneously improve glucose metabolism and restore redox balance.

Among dietary components, polyphenols have attracted considerable attention because of their pleiotropic biological effects. Naturally present in plant-based foods, polyphenols may modulate signaling pathways involved in glucose metabolism, oxidative stress, inflammation, endothelial function, and mitochondrial homeostasis. The Mediterranean diet, characterized by a high intake of fruits, vegetables, whole grains, legumes, nuts, olive oil, and other plant-based foods, represents a particularly rich source of dietary polyphenols [7]. However, evidence regarding the metabolic effects of polyphenols remains heterogeneous because of differences in study populations, intervention types, doses, and study duration [8,9,10]. Moreover, many intervention studies have focused on isolated supplements or extracts rather than polyphenol-rich foods incorporated into a dietary pattern.

Importantly, intervention studies conducted specifically in individuals with prediabetes remain limited, and few have simultaneously assessed glycemic control and oxidative–antioxidant balance [11]. Given that impaired redox homeostasis may contribute to metabolic deterioration and cardiometabolic complications in prediabetes, the combined assessment of glucose metabolism and redox biomarkers may provide a more comprehensive understanding of the effects of polyphenol-rich dietary interventions.

Therefore, the aim of this study was to evaluate whether adding daily chokeberry juice to Mediterranean diet recommendations improves glycemic control and redox homeostasis in women with prediabetes.

## 2. Materials and Methods

### 2.1. Study Participants

**Figure 1:**
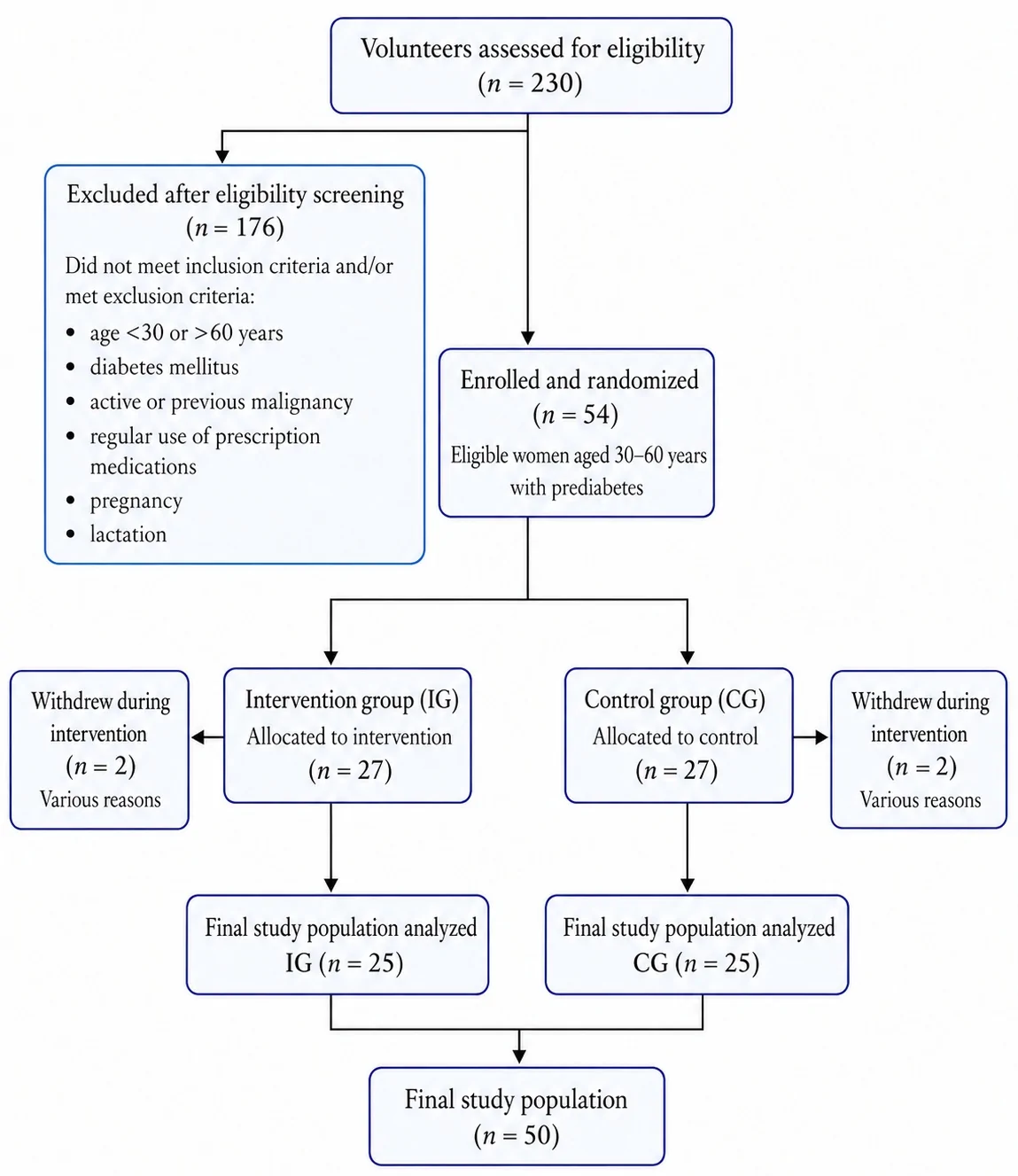
Flow-chart of study participants.

Participants for the present randomized substudy were drawn from a larger mixed-sex cohort enrolled in the single-group parent project registered as NCT06435130. The parent record describes the broader multi-phase study, whereas this manuscript reports a nested two-arm randomized comparison restricted to women with prediabetes who completed a 12-week juice-only protocol; no separate registration was created for this substudy. Women aged 30–60 years with prediabetes were eligible for inclusion. None of the enrolled participants had diabetes; accordingly, diabetes severity and duration were not applicable. The time since first identification of prediabetes was not collected. Exclusion criteria included age <30 or >60 years, diabetes mellitus, active or previous malignancy, regular use of prescription medications, pregnancy, and lactation. A total of 230 women volunteered to participate in the study. Following eligibility screening, 54 women met the inclusion criteria and were enrolled in this study. Participants were randomly assigned in a 1:1 ratio to either the intervention group (IG) (*n* = 27) or the control group (CG) (*n* = 27) using a computer-generated randomization sequence. Allocation concealment was ensured by an independent researcher who generated the randomization sequence, while participant recruitment was conducted by a separate investigator who remained unaware of the allocation sequence until participants had been enrolled. During the intervention, two participants in the IG withdrew because of the astringent taste of chokeberry juice, and two participants in the CG withdrew for personal reasons. Consequently, the final study population comprised 50 participants, with 25 participants in the intervention group and 25 in the control group. A flow-chart of study participants is shown in Figure 1. The analyses reported here were based on the 50 participants who completed both baseline and 12-week assessments.

### 2.2. Prediabetes Criteria

Prediabetes was defined according to the American Diabetes Association criteria as an HbA1c level of 5.7–6.4% (39–47 mmol/mol), a fasting plasma glucose level (FG) of 100–125 mg/dL (5.6–6.9 mmol/L; impaired fasting glucose, IFG), or a 2 h plasma glucose (2h-PG) concentration of 140–199 mg/dL (7.8–11.0 mmol/L; impaired glucose tolerance, IGT) during a 75 g oral glucose tolerance test (OGTT) [12].

### 2.3. Data Collection and Assays

Basic demographic characteristics were collected using a self-administered questionnaire. Physical activity was assessed using the short form of the International Physical Activity Questionnaire (IPAQ) [13]. Based on responses to the seven questionnaire items, participants were classified into three physical activity categories: low, moderate, or high. Anthropometric measurements, including body weight and height, were performed, and body mass index (BMI) was calculated as body weight (kg) divided by height squared (m^2^). Fasting venous blood samples were collected from all participants and stored at −80 °C until analysis. FG and HbA1c were measured, and OGTT was performed in a certified laboratory using standardized methods. FG was determined using a Cobas c111 analyzer (Roche Diagnostics, Meylan, France), and HbA1c was measured by high-performance liquid chromatography (HPLC) on a D-10 analyzer (Bio-Rad, Hercules, CA, USA). Total antioxidant status (TAS) and total oxidant status (TOS) were measured spectrophotometrically using the ImAnOx (TAS/TAC) and PerOx (TOS/TOC) assay kits (Immundiagnostik AG, Bensheim, Germany) on an Infinite M200 Pro microplate reader (Tecan, Männedorf, Switzerland). The oxidative stress index (OSI) was calculated as the TOS-to-TAS ratio.

### 2.4. Dietary Assessment

Dietary intake was assessed at baseline and after the 12-week intervention using three 24 h dietary recalls (two weekdays and one weekend day). Portion sizes were estimated using a photographic atlas of commonly consumed Polish foods and dishes [14]. Energy and nutrient intakes were calculated using the Diet 6.0 software, based on a database of over 3000 foods, dishes, and dietary supplements commonly consumed in Poland. Dietary total polyphenol intake (DTPI) was estimated by multiplying the daily intake of each food item by its polyphenol content using the Phenol-Explorer database and was expressed in mg GAE (gallic acid equivalents) [15]. Dietary total antioxidant capacity (DTAC) was estimated by multiplying the daily intake of each food item by its antioxidant capacity measured by the FRAP (ferric reducing antioxidant power) method and was expressed in mmol [16]. DTPI and DTAC derived from dietary recalls represent background habitual dietary intake and exclude the study product provided to the intervention group. Accordingly, these estimates characterize background dietary exposure and do not represent total intervention-related polyphenol or antioxidant exposure in the IG. Because three 24 h dietary recalls may not fully reflect typical intake and are subject to errors associated with the processes of recall and reporting, these estimates should be treated as approximate measures of food intake. self-reported 24 h recalls as a limitation because they are subject to recall and reporting bias and may not fully capture usual intake.

### 2.5. Dietary Intervention

The dietary intervention consisted of the daily consumption of 100 mL of commercially available organic chokeberry (*Aronia melanocarpa*) juice by participants in the intervention group. According to the product label, the juice was freshly pressed from fruit, not produced from concentrate, and pasteurized. Before the intervention, all chokeberry juices available on the retail market were screened, and the product with the highest measured total polyphenol content and antioxidant capacity was selected for the trial. The manufacturer specified room-temperature storage and an expiry date on the package; the juice was stored in accordance with these labelled conditions and used within its stated shelf life. The exact industrial pasteurization parameters were proprietary and were not available to the investigators.

**Figure 2:**
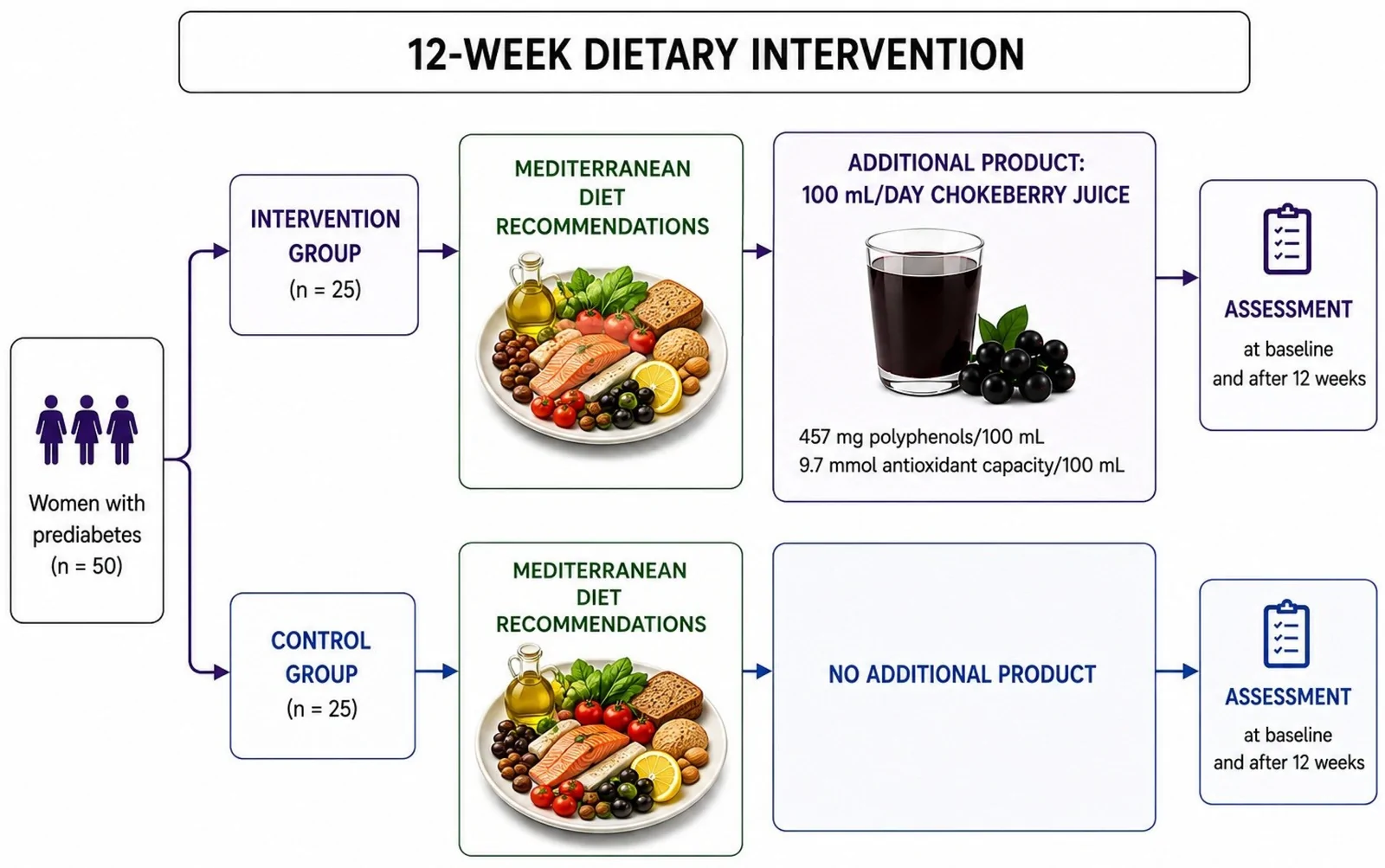
Dietary intervention protocol. Both groups received the same Mediterranean diet recommendations for 12 weeks; the intervention group additionally consumed 100 mL/day of chokeberry juice, whereas the control group received no study product.

The antioxidant capacity and total polyphenol content of the chokeberry juice had been determined in a previous study [17]. Antioxidant capacity, measured using the FRAP assay, was 9.7 mmol/100 mL, and the total polyphenol content, determined using the Folin–Ciocalteu method and expressed as gallic acid equivalents (GAE), was 457 mg GAE/100 mL. Participants in both the intervention and control groups were advised to follow the principles of the Mediterranean diet throughout the 12-week study period and to maintain their usual lifestyle [7,18]. All participants were also instructed to refrain from using dietary supplements. In addition to these dietary recommendations, participants in the intervention group received the study product and were instructed to consume 100 mL of chokeberry juice daily, whereas participants in the control group did not receive the study product. Throughout the intervention period, participants were able to contact the research team by telephone or email if they had any questions or concerns regarding the study protocol. In addition, participants were contacted by telephone once a week to monitor and reinforce adherence to the study recommendations. At the end of the 12-week intervention, all participants self-reported adherence to the study recommendations. The dietary intervention scheme is presented in Figure 2. Accordingly, the randomized between-group contrast represented the addition of chokeberry juice to the same Mediterranean diet recommendations rather than a comparison of two different background diets.

### 2.6. Statistical Analysis

Statistical analyses were performed using STATISTICA software (version 14.1.0.4 PL; TIBCO Software Inc., Palo Alto, CA, USA). Hodges–Lehmann location-shift estimates and their 95% confidence intervals were additionally calculated with the wilcox.test function in R software (version 4.2.2; R Foundation for Statistical Computing, Vienna, Austria). Normality of continuous variables was assessed using the Shapiro–Wilk test. Because the analyzed variables were not normally distributed, continuous data are presented as median and interquartile range (IQR). Intervention-effect analyses were conducted using a complete-case approach and included participants with available measurements at both baseline and 12 weeks (*n* = 50). This pilot substudy used the available eligible sample; no formal a priori sample-size or statistical-power calculation was performed. No prospective hierarchy of primary and secondary endpoints was specified, and all reported glycemic and redox-related outcomes should therefore be interpreted as exploratory.

Baseline characteristics of the intervention group (IG) and control group (CG) were compared using the Mann–Whitney U test for continuous variables and Fisher’s exact test for categorical variables. Within-group changes from baseline to the 12-week follow-up were assessed using the Wilcoxon signed-rank test and were treated as supplementary analyses.

To evaluate the intervention effect, individual changes were calculated as Δ = 12-week value − baseline value, and between-group differences in Δ were assessed using the Mann–Whitney U test. These between-group comparisons constituted the main analyses of intervention effects. The magnitude of between-group differences in change was quantified using Cliff’s delta (δ), a non-parametric measure of effect size.

To express treatment effects in the original measurement units, between-group differences in individual changes were additionally summarized using the Hodges–Lehmann location-shift estimator, calculated as the median of all pairwise differences between change scores in the intervention and control groups (ΔIG − ΔCG), together with a rank-based 95% confidence interval. Negative estimates indicate a greater reduction in the intervention group, whereas positive estimates indicate a greater increase.

To account for multiple testing in this exploratory analysis, *p* values for intervention effects were adjusted using the Benjamini–Hochberg procedure to control the false discovery rate (FDR) within two conceptually distinct outcome families: glycemic outcomes (FG, HbA1c, and 2h-PG) and redox-related outcomes (TOS, TAS, and OSI). These groupings were defined for multiplicity control in the present analysis and do not represent a prospectively prespecified primary–secondary endpoint hierarchy. The same correction was applied to supplementary within-group comparisons within each outcome family. BMI and dietary variables were not included in either FDR outcome family. The reported 95% confidence intervals were nominal and were not adjusted for multiple comparisons.

All tests were two-sided. An FDR-adjusted *p* value < 0.05 was considered statistically significant for analyses subject to multiple-testing correction; otherwise, *p* < 0.05 was considered statistically significant.

## 3. Results

Baseline characteristics of the intervention and control groups are presented in Table 1. No statistically significant between-group differences were detected for age, smoking status, physical activity, BMI, FG, HbA1c, 2h-PG, TOS, TAS, or OSI at baseline.

Table 2 presents habitual dietary intake, excluding the study product, in the intervention and control groups at baseline and after 12 weeks. Total energy intake and intakes of protein, total fat, saturated fatty acids, carbohydrates, monosaccharides, iron, zinc, calcium, vitamin D, and vitamin A did not change significantly within either group. Significant within-group increases in MUFA, dietary fiber, magnesium, vitamin C, vitamin E, dietary total polyphenol intake (DTPI), and dietary total antioxidant capacity (DTAC) were observed in both groups. At 12 weeks, no statistically significant between-group differences were detected for any of the analyzed dietary variables.

**Figure 3:**
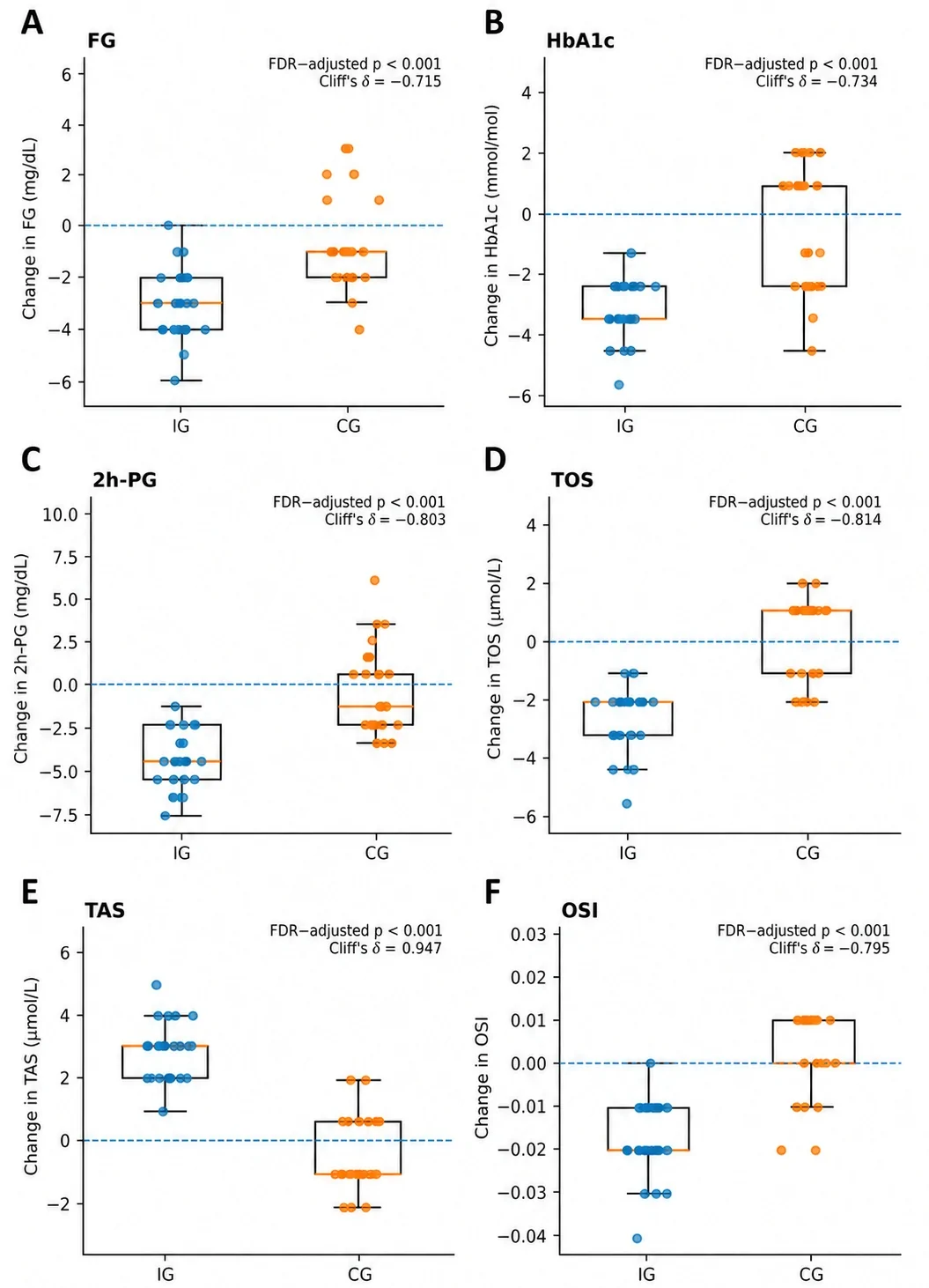
Changes in glycemic and redox parameters after 3 months in the intervention group (IG) and control group (CG): (**A**)—FG, (**B**)—HbA1c, (**C**)—2h-PG, (**D**)—TOS, (**E**)—TAS, (**F**)—OSI. Individual changes are shown as Δ = 3-month value − baseline value, together with the median and IQR. The dashed line indicates Δ = 0. Between-group differences were assessed using the Mann–Whitney U test with FDR correction; *p* < 0.001 for all parameters. Effect sizes are reported as Cliff’s δ.

Table 3 and Figure 3 present changes in BMI, glycemic and redox-related parameters from baseline to the 3-month follow-up in the intervention group and control group.

Following the 12-week intervention, changes in all glycemic parameters differed significantly between the IG and CG. Median changes in FG, HbA1c, and 2h-PG in the IG were −3 (IQR: −4 to −2) mg/dL, −3 (−3 to −2) mmol/mol, and −4 (−5 to −2) mg/dL, respectively, compared with −1 (−2 to −1) mg/dL, 1 (−2 to 1) mmol/mol, and −1 (−2 to 1) mg/dL in the CG. All between-group differences remained statistically significant after Benjamini–Hochberg correction (FDR-adjusted *p* < 0.001). The corresponding Cliff’s delta values were −0.715 for FG, −0.734 for HbA1c, and −0.803 for 2h-PG, indicating large separation between the distributions of individual changes. Supplementary within-group analyses showed significant improvements in all three glycemic parameters in the IG (FDR-adjusted *p* < 0.001), whereas no significant changes were observed in the CG.

Changes in all redox-related parameters also differed significantly between the IG and CG. In the IG, TOS decreased by a median of −2 (IQR: −3 to −2), TAS increased by 3 (2 to 3), and OSI decreased by −0.02 (−0.02 to −0.01). In the CG, the corresponding median changes were 1 (−1 to 1), −1 (−1 to 1), and 0 (0 to 0.01), respectively. All between-group differences remained statistically significant after Benjamini–Hochberg correction (FDR-adjusted *p* < 0.001). The corresponding Cliff’s delta values were −0.814 for TOS, 0.947 for TAS, and −0.795 for OSI, indicating large separation between the distributions of individual changes. BMI did not change significantly during the intervention. The median individual change was −0.4 kg/m^2^ (IQR: −1.0 to 1.0) in the intervention group and 0.6 kg/m^2^ (IQR: −0.6 to 0.7) in the control group, with no significant between-group difference (*p* = 0.310; Cliff’s δ = −0.168).

The Hodges–Lehmann estimates of the between-group differences in change (ΔIG − ΔCG) were −2 mg/dL (95% CI: −3 to −1) for FG, −3 mmol/mol (95% CI: −4 to −1) for HbA1c, and −3 mg/dL (95% CI: −5 to −2) for 2h-PG. Although all confidence intervals excluded zero, the absolute between-group differences in glycemic outcomes were modest.

For the redox-related outcomes, the corresponding estimates were −3 µmol/L (95% CI: −3 to −2) for TOS, 3 µmol/L (95% CI: 3 to 4) for TAS, and −0.02 (95% CI: −0.02 to −0.01) for OSI. Thus, the intervention was associated with lower oxidant status and OSI and higher antioxidant status relative to the control condition.

## 4. Discussion

The present randomized controlled trial found that, in women with prediabetes advised to follow Mediterranean diet principles, adding 100 mL/day of chokeberry (*Aronia melanocarpa*) juice for 12 weeks was associated with greater improvements in glycemic control and systemic redox-related indices than Mediterranean diet recommendations alone. Compared with the control group, the intervention group showed greater reductions in FG, HbA1c, 2h-PG, TOS, and OSI and a greater increase in TAS. All between-group differences remained significant after FDR correction and were accompanied by large Cliff’s delta values. However, the absolute changes in glycemic variables were modest, and large distribution-based effect sizes should not be interpreted as evidence of established long-term clinical benefit. The clinical relevance and durability of these changes require confirmation in larger and longer studies.

The improvement in glycemic control observed in the present study is consistent with findings from dietary interventions conducted in adults with prediabetes. A subsequent analysis of the same strawberry crossover trial showed that 12 weeks of strawberry consumption increased serum superoxide dismutase activity, glutathione concentrations, and antioxidant capacity while also improving fasting glucose [11,19]. Similar to the present study, the intervention was based on a polyphenol-rich food product rather than an isolated bioactive compound and lasted approximately three months.

Evidence from randomized trials with purified anthocyanins also supports a potential effect on glucose regulation, although findings are not uniform. In a 12-week randomized placebo-controlled trial involving 160 adults with prediabetes or early untreated diabetes, 320 mg/day of purified anthocyanins resulted in a modest reduction in HbA1c compared with placebo [20]. A subsequent analysis of participants with fasting hyperglycemia from the same trial showed a reduction in fasting glucose of approximately 0.4 mmol/L relative to placebo [21]. More recently, Yu et al. reported that 160 mg/day of anthocyanins for 12 weeks increased the rate of reversion from impaired glucose tolerance to normal glucose tolerance (55.9% vs. 29.4%) and improved the Matsuda insulin sensitivity index, whereas the primary outcome of beta-cell function did not differ significantly between groups [22]. These observations suggest that improved insulin sensitivity may contribute to the glycemic effects of anthocyanins, although insulin sensitivity and beta-cell function were not directly assessed in the present study.

Further support comes from a recent large multicenter trial of Totum-63, a polyphenol-rich blend of plant extracts, in participants with prediabetes or early-stage type 2 diabetes. After six months, fasting plasma glucose was significantly reduced compared with placebo in the double-blind treatment arm, with an estimated placebo-adjusted difference of −3.8 mg/dL; a larger reduction was observed in the open-label dosing arm [23]. Collectively, these studies suggest that modest improvements in glycemic control may occur across different sources and formulations of dietary polyphenols, although the magnitude of response varies according to the intervention, metabolic phenotype, dose, and study design.

The additional product used in the present study was chokeberry juice, and the available clinical evidence for Aronia remains heterogeneous. Chamberlin et al. reported that consumption of 100 mL of *Aronia melanocarpa* juice daily for 30 days reduced postprandial glucose and altered serum and fecal metabolites related to carbon and lipid metabolism [24], although the trial included only 14 participants. In adults with prediabetes, Park et al. found that a 12-week preparation containing Aronia together with red ginseng, shiitake mushroom, and nattokinase did not significantly affect glucose concentrations during OGTT, although fasting insulin and HOMA-IR improved [25]. Similarly, an eight-week crossover trial of fermented and non-fermented Aronia extracts in participants with type 2 diabetes showed no meaningful effects on conventional glycemic outcomes [26]. A recent meta-analysis of seven randomized trials likewise found no significant overall effect of Aronia consumption on fasting glucose [27]. Thus, the present randomized comparison is consistent with an additional effect of chokeberry juice as a whole food product, but it does not establish that the observed effects were caused specifically by polyphenols, anthocyanins, or any single constituent of the juice. Current human evidence does not establish that regular consumption of dietary phenolic compounds causes pharmacological-type tolerance or a progressive loss of biological responsiveness. A 24-week randomized placebo-controlled trial of 320 mg/day of purified anthocyanins reported that supplementation was safe and well tolerated, without a signal indicating progressive loss of response [28]. Nevertheless, long-term trials specifically designed to test attenuation of metabolic or redox responses over time are scarce. The present 12-week study cannot determine whether effects persist, plateau, or diminish with substantially longer exposure; therefore, the findings should not be interpreted as supporting indefinite supplementation or as defining a long-term dose. Chokeberry juice should be regarded as a food product used within a balanced dietary pattern rather than as a substitute for preventive or pharmacological care.

An important feature of the present design is that both groups received the same Mediterranean diet recommendations. Dietary recalls excluding the study product showed significant within-group increases in MUFA, dietary fiber, magnesium, vitamins C and E, DTPI, and DTAC in both groups, and no statistically significant between-group differences were detected in the reported dietary variables at 12 weeks. This pattern is compatible with a shared shift toward a Mediterranean-style dietary pattern. However, the dietary analysis compared post-intervention values between groups rather than directly testing between-group differences in dietary change scores; therefore, equivalence of background dietary changes cannot be inferred. Components of the Mediterranean diet, including nuts and other sources of unsaturated fatty acids and polyphenols, may themselves influence glycemic control, although intervention findings are not uniform [29,30]. Accordingly, the randomized contrast is most appropriately interpreted as the effect associated with adding chokeberry juice within the context of shared Mediterranean diet recommendations, rather than as the isolated effect of dietary polyphenols.

The simultaneous improvement in redox-related indices is another relevant finding. TOS and OSI decreased, whereas TAS increased in the intervention group relative to the control group. A subsequent analysis from the strawberry crossover trial reported by Groven [11] and Basu [19] showed that 12 weeks of strawberry consumption increased serum superoxide dismutase activity, glutathione concentrations, and antioxidant capacity while also improving fasting glucose in adults with prediabetes. Thus, concurrent changes in glycemic and antioxidant-related outcomes have also been observed in another berry-based food intervention.

Additional evidence is provided by a randomized double-blind trial of hydroxytyrosol in adults with overweight and prediabetes. Supplementation with 15 mg/day for 16 weeks reduced oxidized LDL, protein carbonyls, and 8-OHdG compared with placebo and prevented decreases in TAS and glutathione peroxidase activity [31]. Similarly, in patients with type 2 diabetes, six weeks of pomegranate juice consumption increased total serum antioxidant capacity and reduced indices of LDL oxidation [32]. Although these studies differ in participant characteristics and polyphenol sources, they support the concept that polyphenol-rich foods and phenolic compounds may favorably influence systemic oxidative-antioxidant status. At the same time, TAS, TOS, and the derived OSI are global redox-related indices and do not identify specific molecular targets or pathways of oxidative damage.

The relationship between the glycemic and redox effects observed in the present study is biologically plausible, but a causal mechanism cannot be established from these data. Polyphenols may influence glucose metabolism and redox homeostasis through several mechanisms, including modulation of intestinal glucose handling, insulin signaling, endogenous antioxidant defenses, inflammation, and mitochondrial and endothelial function [8,9,10]. Of particular relevance, the recent anthocyanin trial in participants with impaired glucose tolerance showed that improved insulin sensitivity occurred together with a reduction in advanced glycation end products, with changes in these variables being correlated [22]. Nevertheless, the present study did not assess insulin, HOMA-IR, OGTT-derived indices of insulin sensitivity, beta-cell function, or specific molecular markers of oxidative damage. Therefore, it cannot be determined whether the improvement in redox-related indices contributed mechanistically to the glycemic changes or represented a parallel response to the intervention.

Taken together, the randomized design, shared Mediterranean diet recommendations, similar reported post-intervention background dietary intake, and lack of a significant between-group difference in BMI change support an association between the observed between-group effects and the addition of chokeberry juice. Nevertheless, differential changes in background diet cannot be excluded completely because dietary change scores were not directly compared between groups. Moreover, because the intervention used whole juice, the observed effects cannot be separated into polyphenol-specific and non-polyphenol components of the food matrix. The control group did not receive a matched placebo, and blinding of participants and investigators involved in delivering the intervention was not feasible. Consequently, performance bias and nonspecific behavioral or expectancy effects cannot be excluded; although the outcomes were objective laboratory measurements, detection bias cannot be fully ruled out.

### Strengths and Limitations

The strengths of this study include its randomized controlled design, baseline comparability of the groups, and the use of the same Mediterranean diet recommendations in both study arms, which provided an active dietary background against which the additional effect of chokeberry juice could be evaluated. The juice was characterized in terms of total polyphenol content and antioxidant capacity. Both glycemic and redox-related outcomes were assessed; the primary analysis was based on between-group differences in individual changes, multiple testing was addressed using false discovery rate correction, and effect sizes were reported. Attrition was low and balanced between groups.

Several limitations should be considered. The relatively small sample included only women aged 30–60 years, limiting generalizability. The 12-week intervention does not allow conclusions regarding the long-term persistence of the observed effects or progression to type 2 diabetes. The study was not placebo-controlled, and adherence to the Mediterranean diet and juice intervention was based primarily on participant report. Dietary intake was assessed using self-reported 24 h recalls, which are subject to recall and reporting bias. In addition, TAS, TOS, and OSI provide only global information on redox status. Insulin, HOMA-IR, beta-cell function, and specific molecular markers of oxidative damage were not assessed, limiting mechanistic interpretation. A validated Mediterranean-diet adherence score was not collected; consequently, adherence to the recommended dietary pattern could not be quantified directly or analyzed as a change score. The duration of prediabetes before enrollment was not recorded. Furthermore, the 12-week follow-up was insufficient to assess long-term safety or whether metabolic and redox responses to phenolic compounds might attenuate, remain stable, or increase during prolonged consumption. Because chokeberry juice is a complex food matrix, the observed effects cannot be attributed specifically to polyphenols or anthocyanins. Future studies should include larger and more diverse populations, longer follow-up, a matched placebo where feasible, objective biomarkers of adherence and polyphenol exposure, and more comprehensive metabolic and redox profiling. A validated Mediterranean-diet adherence score was not collected; consequently, adherence to the recommended dietary pattern could not be quantified directly or analyzed as a change score. The duration of prediabetes before enrollment was not recorded. Furthermore, the 12-week follow-up was insufficient to assess long-term safety or whether metabolic and redox responses to phenolic compounds might attenuate, remain stable, or increase during prolonged consumption.

## 5. Conclusions

In women with prediabetes receiving Mediterranean diet recommendations, the addition of 100 mL/day of chokeberry juice for 12 weeks was associated with modest but statistically significant improvements in FG, HbA1c, 2h-PG, and systemic redox-related indices compared with Mediterranean diet recommendations alone. These findings suggest an additional effect of chokeberry juice within the shared dietary intervention; however, they do not allow the observed effects to be attributed specifically to polyphenols or establish long-term clinical benefit. The small, female-only sample, short follow-up, complete-case analysis, lack of placebo control, reliance on self-reported adherence, and absence of mechanistic biomarkers limit the strength and generalizability of the findings. Larger, longer, placebo-controlled trials are needed to confirm the clinical relevance and durability of these effects and to clarify the underlying mechanisms.

## Figures and Tables

**Table 1:** Baseline characteristics of the intervention and control groups.

Variables	Total	Intervention Group	Control Group	*p*
Sex, women, *n* (%)	50 (100)	25 (100)	25 (100)	-
Age (years), Me (IQR)	44.5 (37, 52)	45.2 (38, 53)	43.9 (39, 51)	0.345
Current smoking, *n* (%)	9 (18)	5 (20)	4 (16)	1.000
Physical activity, *n* (%)				
high	0 (0)	0 (0)	0 (0)	1.000
moderate	17 (34)	9 (36)	8 (32)	
low	33 (66)	16 (64)	17 (68)	
BMI (kg/m^2^), Me (IQR)	25.7 (24.4, 26.4)	25.8 (24.6, 26.7)	25.4 (24.1, 26.3)	0.455
FG (mg/dL), Me (IQR)	112.5 (107, 120)	112 (107, 120)	113 (108, 118)	0.954
HbA1c (mmol/mol), Me (IQR)	44 (42, 45)	44 (42, 45)	44 (42, 45)	0.908
2h-PG (mg/dL), Me (IQR)	163.5 (155, 180)	164 (151, 183)	163 (157, 180)	0.744
TOS (µmol/L), Me (IQR)	97.3 (87.3, 105.2)	96.2 (85.3, 105.4)	99.2 (94.5, 104.7)	0.449
TAS (µmol/L), Me (IQR)	207.1 (189.3, 215.8)	213.2 (195.1, 215.4)	202.9 (188.4, 223.2)	0.509
OSI (TOS/TAS)	0.47 (0.45, 0.5)	0.47 (0.42, 0.5)	0.48 (0.46, 0.5)	0.263

*n*—number, Me—median, IQR—interquartile range, BMI—body mass index, FG—fasting glucose, HbA1c—glycated hemoglobin, 2h-PG—2 h plasma glucose concentration in OGTT (oral glucose tolerance test), TOS—total oxidant status, TAS—total antioxidant status, OSI—oxidative stress index.

**Table 2:** Habitual dietary intake, excluding study products, at baseline and after a 3-month Mediterranean diet intervention in both the intervention and control groups.

Variables	Intervention Group		*P_IG_*	Control Group		*P_CG_*	*P_IG/CG after 3m_*
	Baseline	After 3-Month		Baseline	After 3-Month		
Energy (kcal)	2134 (1745, 2437)	2212 (1695, 2511)	0.534	2012 (1634, 2524)	2132 (1598, 2612)	0.436	0.376
Protein (g)	76 (65, 91)	80 (72, 95)	0.342	81 (71, 98)	84 (69, 97)	0.389	0.112
Fat (g)	79 (64, 89)	76 (62, 92)	0.428	77 (59, 93)	80 (62, 94)	0.234	0.214
SFA (g)	27 (18, 35)	31 (17, 37)	0.285	30 (16, 41)	28 (16, 36)	0.311	0.114
MUFA (g)	31 (27, 38)	38 (29, 45)	**0.002**	30 (26, 36)	39 (30, 46)	**0.001**	0.487
Carbohydrates (g)	243 (182, 284)	238 (194, 312)	0.384	238 (191, 298)	241 (201, 312)	0.287	0.467
Monosaccharides (g)	68 (47, 84)	70 (51, 86)	0.225	71 (45, 91)	69 (48, 92)	0.299	0.532
Dietary fiber (g)	18 (14, 22)	26 (21, 31)	**0.011**	20 (16, 26)	27 (19, 32)	**0.009**	0.545
Magnesium (mg)	286 (268, 324)	325 (291, 362)	**0.021**	301 (272, 341)	334 (283, 353)	**0.032**	0.342
Iron (mg)	12 (8, 14)	13 (9, 15)	0.059	13 (8, 14)	14 (9, 16)	0.065	0.152
Zinc (mg)	11 (8, 12)	12 (9, 14)	0.086	10 (7, 13)	12 (7, 14)	0.092	0.247
Calcium (mg)	711 (678, 865)	732 (679, 891)	0.233	697 (654, 789)	715 (687, 811)	0.256	0.364
Vitamin C (mg)	84 (65, 122)	105 (78, 151)	**0.003**	81 (62, 112)	101 (75, 142)	**0.005**	0.123
Vitamin D (µg)	4.3 (3.7, 4.6)	4.4 (3.7, 4.8)	0.287	4.5 (3.8, 5.1)	4.6 (3.7, 5.1)	0.212	0.231
Vitamin A (µg)	831 (743, 924)	847 (734, 942)	0.311	822 (719, 896)	839 (711, 921)	0.295	0.312
Vitamin E (mg)	11 (8, 13)	13 (9, 15)	**0.029**	10 (7, 14)	14 (9, 16)	**0.021**	0.074
DTPI (mg GAE)	1843 (1523, 2345)	2095 (1689, 2578)	**0.036**	1811 (1554, 2412)	2032 (1587, 2399)	**0.028**	0.342
DTAC (mmol)	12 (8, 16)	14 (10, 18)	**0.018**	13 (9, 15)	15 (9, 17)	**0.024**	0.085

Results are presented as Me (IQR) (median and interquartile range). Bold font indicates statistically significant results. SFA—saturated fatty acids, MUFA—monounsaturated fatty acids, DTPI—dietary total polyphenol intake, DTAC—dietary total antioxidant capacity, GAE—gallic acid.

**Table 3:** Baseline values and changes in BMI, glycemic, and redox-related parameters after the 3-month intervention in the intervention and control groups.

Variables	Intervention Group		ΔIG	Control Group		ΔCG	*p* for ΔIG vs. CG	FDR- Adjusted *p*	H–L est. for ΔIG − ΔCG (95% CI)	Cliff’s δ
	Baseline	After 3-Month		Baseline	After 3-Month					
BMI (kg/m^2^)	25.8 (24.6–26.7)	25.9 (23.8–26.9)	−0.4 (−1 to 1)	25.4 (24.1–26.3)	25.8 (24.4–26.4)	0.6 (−0.6 to 0.7)	0.310	-		−0.168
FG (mg/dL)	112 (107, 120)	109 (103, 117)	−3 (−4 to −2)	113 (108, 118)	112 (108, 118)	−1 (−2 to −1)	<0.001	**<0.001**	−2 (−3 to −1)	−0.715
HbA1c (mmol/mol)	44 (42, 45)	41 (39, 43)	−3 (−3 to −2)	44 (42, 45)	43 (42, 45)	1 (−2 to 1)	<0.001	**<0.001**	−3 (−4 to −1)	−0.734
2h-PG (mg/dL)	164 (151, 183)	159 (148, 179)	−4 (−5 to −2)	163 (157, 180)	162 (157, 178)	−1 (−2 to 1)	<0.001	**<0.001**	−3 (−5 to −2)	−0.803
TOS (µmol/L)	96 (85–105)	94 (82–103)	−2 (−3 to −2)	99 (94–105)	99 (94–105)	1 (−1 to 1)	<0.001	**<0.001**	−3 (−3 to −2)	−0.814
TAS (µmol/L)	213 (195–215)	216 (197–218)	3 (2 to 3)	203 (188–223)	203 (188–222)	−1 (−1 to 1)	<0.001	**<0.001**	3 (3 to 4)	0.947
OSI (TOS/TAS)	0.47 (0.42–0.50)	0.45 (0.41–0.48)	−0.02 (−0.02 to −0.01)	0.48 (0.46–0.50)	0.48 (0.46–0.51)	0 (0 to 0.01)	<0.001	**<0.001**	−0.02 (−0.02 to −0.01)	−0.795

Data are presented as median (IQR). Changes were calculated as Δ = 12-week value − baseline value and compared between groups using the Mann–Whitney U test. *p* values were adjusted using the Benjamini–Hochberg FDR procedure separately for glycemic (FG, HbA1c, 2h-PG) and redox-related outcomes (TOS, TAS, OSI). BMI was not included in the FDR correction. Cliff’s delta (δ) was used as the effect-size measure. FG—fasting glucose, HbA1c—glycated hemoglobin, 2h-PG—2 h plasma glucose, TOS—total oxidant status, TAS—total antioxidant status, OSI—oxidative stress index, BMI—body mass index, H-L est.—Hodges–Lehmann estimate. Bold font indicates statistically significant results.

## Data Availability

The original contributions presented in this study are included in the article. Further inquiries can be directed to the corresponding author.

## References

[1] American Diabetes Association Professional Practice Committee for Diabetes (2026). 3. Prevention or Delay of Diabetes and Associated Comorbidities: Standards of Care in Diabetes-2026. Diabetes Care.

[2] Sies H., Jones D.P. (2020). Reactive oxygen species (ROS) as pleiotropic physiological signalling agents. Nat. Rev. Mol. Cell Biol..

[3] Dinić S., Arambašić Jovanović J., Uskoković A., Mihailović M., Grdović N., Tolić A., Rajić J., Đorđević M., Vidaković M. (2022). Oxidative stress-mediated beta cell death and dysfunction as a target for diabetes management. Front. Endocrinol..

[4] Veluthakal R., Esparza D., Hoolachan J.M., Balakrishnan R., Ahn M., Oh E., Jayasena C.S., Thurmond D.C. (2024). Mitochondrial dysfunction, oxidative stress, and inter-organ miscommunications in T2D progression. Int. J. Mol. Sci..

[5] Crandall J.P., Dabelea D., Knowler W.C., Nathan D.M., Temprosa M., DPP Research Group (2025). The diabetes prevention program and its outcomes study: NIDDK’s journey into the prevention of type 2 diabetes and its public health impact. Diabetes Care.

[6] Zujko M.E., Rożniata M., Zujko K. (2021). Individual diet modification reduces the metabolic syndrome in patients before pharmacological treatment. Nutrients.

[7] Sofi F., Martini D., Angelino D., Cairella G., Campanozzi A., Danesi F., Dinu M., Erba D., Iacoviello L., Pellegrini N. (2025). Mediterranean diet: Why a new pyramid? An updated representation of the traditional Mediterranean diet by the Italian Society of Human Nutrition (SINU). Nutr. Metab. Cardiovasc. Dis..

[8] Shahidi F., Danielski R. (2024). Review on the role of polyphenols in preventing and treating type 2 diabetes: Evidence from in vitro and in vivo studies. Nutrients.

[9] Saad A.M., Mohammed D.M., Alkafaas S.S., Ghosh S., Negm S.H., Salem H.M., Fahmy M.A., Semary H.E., Ibrahim E.H., AbuQamar S.F. (2025). Dietary polyphenols and human health: Sources, biological activities, nutritional and immunological aspects, and bioavailability—A comprehensive review. Front. Immunol..

[10] Martiniakova M., Sarocka A., Penzes N., Biro R., Kovacova V., Mondockova V., Sevcikova A., Ciernikova S., Omelka R. (2025). Protective role of dietary polyphenols in the management and treatment of type 2 diabetes mellitus. Nutrients.

[11] Groven S., Devillez P., Scofield R.H., Champion A., Izuora K., Basu A. (2025). Dietary strawberries improve serum antioxidant profiles in adults with prediabetes: A 28-Week randomized controlled crossover trial. Antioxidants.

[12] American Diabetes Association Professional Practice Committee for Diabetes (2026). 2. Diagnosis and Classification of Diabetes: Standards of Care in Diabetes-2026. Diabetes Care.

[13] Biernat E., Stupnicki R., Gajewski A.K. (2007). Międzynarodowy Kwestionariusz Aktywności Fizycznej (IPAQ)—Wersja Polska [International Physical Activity Questionnaire (IPAQ)—Polish Version]. Wychow. Fiz. Sport [Phys. Educ. Sport].

[14] Szponar L., Wolnicka K., Rychlik E. (2000). Album Fotografii Produktow I Potraw [Album of Photographs of Food Products and Dishes].

[15] Neveu V., Perez-Jiménez J., Vos F., Crespy V., du Chaffaut L., Mennen L., Knox C., Eisner R., Cruz J., Wishart D. (2010). Phenol-Explorer: An online comprehensive database on polyphenol contents in foods. Database.

[16] Carlsen M.H., Halvorsen B.L., Holte K., Bøhn S.K., Dragland S., Sampson L., Willey C., Senoo H., Umezono Y., Sanada C. (2010). The total antioxidant content of more than 3100 foods, beverages, spices, herbs and supplements used worldwide. Nutr. J..

[17] Olechno E., Puścion-Jakubik A., Soroczyńska J., Socha K., Cyuńczyk M., Zujko M.E. (2023). Antioxidant properties of chokeberry products—Assessment of the composition of juices and fibers. Foods.

[18] Monteagudo C., Mariscal-Arcas M., Rivas A., Lorenzo-Tovar M.L., Tur J.A., Olea-Serrano F. (2015). Proposal of a Mediterranean diet serving score. PLoS ONE.

[19] Basu A., Hooyman A., Groven S., DeVillez P., Scofield R.H., Ebersole J.L., Champion A., Izuora K. (2025). Strawberries improve insulin resistance and related cardiometabolic markers in adults with prediabetes: A randomized controlled crossover trial. J. Nutr..

[20] Yang L., Qiu Y., Ling W., Liu Z., Yang L., Wang C., Peng X., Wang L., Chen J. (2021). Anthocyanins regulate serum adipsin and visfatin in patients with prediabetes or newly diagnosed diabetes: A randomized controlled trial. Eur. J. Nutr..

[21] Yang L., Liu Z., Ling W., Wang L., Wang C., Ma J., Peng X., Chen J. (2020). Effect of anthocyanins supplementation on serum IGFBP-4 fragments and glycemic control in patients with fasting hyperglycemia: A randomized controlled trial. Diabetes Metab. Syndr. Obes..

[22] Yu L., Amaliyati S., Sun H., Dai J., Jiang Y., Li Z., Dong Z., Zhao L. (2026). Remission of impaired glucose tolerance by anthocyanin supplementation: A randomized, double-blind, and placebo-controlled trial. Am. J. Clin. Nutr..

[23] Chavanelle V., Otero Y.F., Fayat Cazaubiel M., Bard J.M., Pereira B., Sapone V., Bouchard-Mercier A., Bargetto M., Boisseau N., Guigas B. (2026). Totum-63, a plant-based polyphenol-rich extract, in prediabetes or early-stage type 2 diabetes: An international, multicenter, randomized controlled trial. Nat. Commun..

[24] Chamberlin M.L., Peach J.T., Wilson S.M.G., Miller Z.T., Bothner B., Walk S.T., Yeoman C.J., Miles M.P. (2024). Polyphenol-rich *Aronia melanocarpa* fruit beneficially impact cholesterol, glucose, and serum and gut metabolites: A randomized clinical trial. Foods.

[25] Park S., Kim C.J., Ha K.C., Baek H.I., Yang H.J., Kim M.J., Park S.J. (2021). Efficacy and safety of aronia, red ginseng, shiitake mushroom, and nattokinase mixture on insulin resistance in prediabetic adults: A randomized, double-blinded, placebo-controlled trial. Foods.

[26] Christiansen C.B., Jeppesen P.B., Hermansen K., Gregersen S. (2023). Aronia in the type 2 diabetes treatment regimen. Nutrients.

[27] Lohrasbi N., Elahikhah M., Gholizadeh M., Djafari F., Bahreini N., Sheikhhossein F., Askari G., Amini M.R. (2026). The effect of *Aronia melanocarpa* (chokeberry) on body weight and fasting blood sugar: A systematic review and meta-analysis of randomised controlled trials. Endocrinol. Diabetes Metab..

[28] Aarsland D., Khalifa K., Bergland A.K., Soennesyn H., Oppedal K., Holteng L.B.A., Oesterhus R., Nakling A., Jarholm J.A., de Lucia C. (2023). A randomised placebo-controlled study of purified anthocyanins on cognition in individuals at increased risk for dementia. Am. J. Geriatr. Psychiatry.

[29] Ashwini K., Abirami K., Gayathri R., Sasikala S., Sudha V., Shobana S., Jeevan R.G., Krishnaswamy K., Deepika V., Rajalakshmi M. (2025). Effect of premeal pistachio supplementation on cardiometabolic risk factors among Asian Indian adults with prediabetes: A randomized controlled trial. J. Nutr..

[30] Riley T.M., Kris-Etherton P.M., Hart T.L., Petersen K.S. (2024). Intake of pistachios as a nighttime snack has similar effects on short- and longer-term glycemic control compared with education to consume 1–2 carbohydrate exchanges in adults with prediabetes: A 12-wk randomized crossover trial. J. Nutr..

[31] Moratilla-Rivera I., Pérez-Jiménez J., Ramos S., Portillo M.P., Martín M.Á., Mateos R. (2025). Hydroxytyrosol supplementation improves antioxidant and anti-inflammatory status in individuals with overweight and prediabetes: A randomized, double-blind, placebo-controlled parallel trial. Clin. Nutr..

[32] Sohrab G., Ebrahimof S., Sotoudeh G., Neyestani T.R., Angoorani P., Hedayati M., Siasi F. (2017). Effects of pomegranate juice consumption on oxidative stress in patients with type 2 diabetes: A single-blind, randomized clinical trial. Int. J. Food Sci. Nutr..

